# Iconicity can ground the creation of vocal symbols

**DOI:** 10.1098/rsos.150152

**Published:** 2015-08-05

**Authors:** Marcus Perlman, Rick Dale, Gary Lupyan

**Affiliations:** 1 Department of Psychology, University of Wisconsin-Madison, Madison, WI, USA; 2 Cognitive and Information Sciences, University of California, Merced, Merced, CA, USA

**Keywords:** experimental semiotics, iconicity, language evolution, vocalization

## Abstract

Studies of gestural communication systems find that they originate from spontaneously created iconic gestures. Yet, we know little about how people create vocal communication systems, and many have suggested that vocalizations do not afford iconicity beyond trivial instances of onomatopoeia. It is unknown whether people can generate vocal communication systems through a process of iconic creation similar to gestural systems. Here, we examine the creation and development of a rudimentary vocal symbol system in a laboratory setting. Pairs of participants generated novel vocalizations for 18 different meanings in an iterative ‘vocal’ charades communication game. The communicators quickly converged on stable vocalizations, and naive listeners could correctly infer their meanings in subsequent playback experiments. People's ability to guess the meanings of these novel vocalizations was predicted by how close the vocalization was to an iconic ‘meaning template’ we derived from the production data. These results strongly suggest that the meaningfulness of these vocalizations derived from iconicity. Our findings illuminate a mechanism by which iconicity can ground the creation of vocal symbols, analogous to the function of iconicity in gestural communication systems.

## 1. Introduction

In recent decades, scientists have learned a lot about how people create gestural communication systems and the circumstances under which this happens. Observations span the origins of highly sophisticated urban and rural signed languages used by deaf people and their communities [1–5], home sign systems devised by deaf children to communicate with hearing carers [6], and alternative sign languages used by hearing people under different conditions in which speech is disadvantaged or suppressed [7,8]. Experimental studies with hearing participants also show how, when prohibited from speaking, people quickly improvise more language-like means of gesturing [9]. In comparison, we know very little about how people create *vocal* communication systems, including the roughly 7000 languages currently spoken around the world [10].^1^ Here, we examine the creation and development of a rudimentary vocal system in a laboratory setting. Our findings illuminate a mechanism by which iconic vocalizations can ground the creation of vocal symbols, analogous to the function of iconic gestures in gestural communication systems.

The study of gestural systems, including fully conventionalized signed languages, has concluded that they originate largely from spontaneously created *iconic*gestures [12,13]. Iconicity refers to a quality of gestures and other signals that are characterized by a sense of resemblance or correspondence between their form and the meaning they express.^2^ For example, in British Sign Language, the conventional sign ‘hammer’ is formed by simulating the manual action of hammering, and ‘bottle’ is articulated with a curved hand as if handling a bottle, using upward movement to show its shape [14]. These examples also reveal an element of metonymy that is common in iconic gestures: the option to represent concepts by their different characteristic properties, for instance, emphasizing function versus shape. Moreover, iconicity in signs and less conventionalized gestures is not limited to direct enactments and depictions. Iconicity can be highly schematic as in the American Sign Language (ASL) classifier that represents long skinny objects, often a singular person, with an upright index finger [15]. It can also be the source of metaphorical expressions, as in the ASL sign ‘think-penetrate’ (translated as ‘[the subject] finally got the point’), in which the signer uses her hand to depict an idea as an object, and shows it emerging from her forehead and penetrating a barrier represented by her second hand.

Notably, iconicity can be exhibited by signals in various modalities and media. For example, graphical media are often iconic, ranging from detailed drawings to schematic roadway signs [16]. Vocalizations can also be iconic in ways that are comparable to gesture and drawing. The vocal imitation of different environmental sounds is one obvious example of iconicity in vocalization (and presumably the source of conventionalized onomatopoeia in languages). But vocal iconicity can also be highly schematic, as in the elongation of the vowel in ‘a *loooong*time’ to depict the protracted temporal duration of an event [14]. Through metaphorical correspondence across modalities, temporal extension of the vowel can also be used to express spatial extension, as in describing the ‘*loooong*stem’ of a mushroom [17].

Iconicity is theorized to function in the creation of gestural systems by providing an initial connection between a gesture and its meaning [14,18,19]. Iconicity both facilitates the generation of novel forms, and it enables a perceiver to gain a sense meaning without (or at least, with less) previous learning. Building from an iconically grounded connection between form and meaning, gestures become conventionalized and systematic over the course of repeated use and transmission. For example, they become more stereotyped and categorical, and under the right circumstances, they can grow into grammatical systems of varying levels of complexity and conventionalization. For instance, in isolation from other signers, deaf children create iconic gestures that ground the development of symbolic home sign systems that they use (asymmetrically) with hearing adults [6,20]. These gestural systems develop hierarchical levels of organization, along with systematic contrast between gestures. In communities with multiple signers transmitting a growing system over multiple generations—such as in schools for the deaf or rural villages with high incidence of genetic deafness—fully conventionalized, grammatical signed languages can originate from iconic gestures in just a few generations [12,21,22]. Iconic gestures can become systematic on shorter time scales too. Some fairly basic grammatical patterns, such as the use of gesture order to express thematic roles, can emerge quite quickly in the laboratory, such as when hearing adults are tasked to communicate with gestures instead of speech [8].

In addition to gestural systems, studies of communication in graphic media point to a more general process of *iconic creation*, according to which iconic signals generate the raw material for conventionalization, and thereby ground the generation of a communication system. For example, historical investigations of writing systems show that many of the world's written scripts (e.g. Sumerian, early Egyptian, ancient Chinese) originated in more detailed, iconic depictions which over history became conventionalized into an increasingly abstract code [23–25]. More recently, experimental evidence for iconic creation comes from laboratory studies of how graphic communication systems develop. In one set of experiments, participants produced drawings to communicate different items (e.g. Clint Eastwood, soap opera) over multiple blocks [16]. Participants originally produced detailed iconic drawings related to the items. Over repeated rounds, these drawings became increasingly conventionalized and symbolic, but only under conditions in which participants were able to interact with each other and provide feedback.

Given these basic findings in gesture and graphic media, an interesting question is the extent to which people can also generate vocal communication systems by this same process of iconic creation. On the face of it, many scholars have argued that they cannot [12,19,26–29]. Their arguments typically contend that gestures are special in their potential for iconicity, particularly when compared to vocalizations, because of the spatial nature of gestures and their resemblance to manual actions used for manipulating objects [11,12,28]: hence, the pervasive iconicity evident in signed languages. By contrast, vocal communication systems—spoken languages in particular—are often characterized as essentially arbitrary, aside from a few marginal instances of onomatopoeia (i.e. words bearing sound–sound correspondences). For instance, Hockett reasoned that, ‘when a representation of some four-dimensional hunk of life has to be compressed into the single dimension of speech, most iconicity is necessarily squeezed out’ [[10], p. 275] (quoted in [28]). Some experimental support for this line of reasoning comes from a set of studies in which participants communicated an array of actions, objects and emotions using either gestures or non-linguistic vocalizations [19,30]. The results showed that participants performed much better with gestures, leading the authors to tout the superiority of gesture over vocalization for iconic representation. This rationale presents a challenge for scholars aiming to understand the origins of vocal communication systems and how vocal symbols come to be associated with their particular meanings (cf. [31]).

However, as hinted in the examples above, there is considerable evidence indicating that the vocal modality holds more potential for iconicity than is often realized [13,17]. It is widely recognized that vocalizations can be iconic of various environmental sounds in a directly imitative way (e.g. imitating the sounds of animals or cars), and through metonymy and metaphor, these imitative vocalizations can extend to new realms of meaning (e.g. the various senses of ‘splash’) [15]. In addition, the reach of iconicity in vocalization can be greatly expanded through more abstract and schematic connections to meaning. One example comes from research in cross-modal correspondences and synesthesia, which finds that people share deeply rooted associations between certain acoustic and visual dimensions, many of which arise as metaphors in spoken language [32–34]. For instance, people tend to represent dimensions of magnitude according to a common amodal or multimodal representation. These prothetic dimensions include acoustic properties like duration and loudness in association with visual dimensions like brightness and spatial dimensions like size and length. Thus, people are inclined to perceive equivalence between disparate stimuli as a long sound, a loud sound, a bright light, and a big or long object. Studies also show a deep association, detected in infants three to four months of age, between the acoustic dimension of pitch and both visual–spatial height (e.g. high pitch with high spatial position) and visual sharpness (e.g. high pitch with pointedness) [35].

The potential for iconicity in vocalization is also demonstrated by the many studies of sound symbolism, which find that people have a notable degree of agreement in the meanings they associate with different speech sounds, independently of their linguistic meaning [36–38]. One commonly attested example is the association of front vowels (with high-pitched second formants) with small size, and back vowels (with low-pitched second formants) with large size [39]. Another example is the so-called ‘kiki-bouba’ effect [34]: voiceless obstruent consonants alternated with front, closed and unrounded vowels evoke a sense of angular shape, whereas voiced sonorants combined with open, rounded vowels evoke a rounded shape [40]. Importantly, studies have found reliable sound symbolism effects related to a wide variety of semantic domains, including dimensions such as abrupt–continuous, liquid–solid, tight–loose, delicate–rugged, sharp–dull, thick–thin, falling–rising and fast–slow [41,42].

Further evidence for the iconic potential of vocalization comes from cross-linguistic research documenting the widespread existence of rich lexical classes of iconic words in many of the world's languages [36,43,44]. These ideophones are grammatically and phonologically distinct from other classes of words, and they typically convey meaning through the depiction of sensory and motor imagery. For example, in Japanese, the word ‘koron’ refers to a light object rolling once, ‘korokoro’ to a light object rolling repeatedly and ‘gorogoro’ to a heavy object rolling repeatedly. In these cases, reduplication is used for the iconic expression of repetition, and voiced compared to voiceless consonants iconically convey a more massive object. The schematic nature of this iconicity is characteristic of the lexical class, as are the systematic ways in which the words relate to each other. Ideophones are documented to span a range of meanings across different modalities, such as manner of motion, shape, psychological and mental states, texture, size, luminance, distance and temporal aspect. Although this synchronic research cannot uncover the origins of these words, the fact of their existence strongly hints at a process of iconic creation.

Thus, given this considerable potential for iconicity in vocalization, it appears possible that people could establish a vocal communication system through the process of iconic creation, comparable to gesture and drawing. To examine this possibility, we conducted a set of experiments in which we tested the ability of participants to create a simple system of vocal symbols in the laboratory.

## 2. Vocal charades

Pairs of participants played a 10 round game of ‘vocal’ charades. Players took turns producing non-linguistic vocalizations to communicate different meanings as their partner tried to guess the meaning. The set of meanings included nine pairs of antonymic properties spanning a variety of semantic domains: speed, size, length, distance, (vertical) position, quantity, aesthetic and psychological valence. Our goal was to examine whether participants would be able to create iconic vocalizations to successfully communicate each meaning, and then in turn, use these vocalizations to establish a more conventionalized system of vocal symbols. Unavoidably, as in similar semiotics experiments, our participants already spoke a language and thus came well acquainted with symbolic communication. Many previous informative experiments have addressed this issue by tasking participants to communicate in a different modality or medium from speech (e.g. [9,16,45]); here, because of our specific interest in vocalization, we instructed participants to communicate without the words of their spoken language. Our analysis shows they succeeded in observing this constraint.

### 2.1 Methods

#### 2.1.1 Participants

Nine pairs of undergraduate students from the University of California, Santa Cruz participated in the study in exchange for course credit.

#### 2.1.2 Materials

The game was played with nine pairs of antonyms spanning various semantic domains: *attractive, ugly, bad, good, big, small, down, up, far, near, fast, slow, few, many, long, short*, *rough*and *smooth*. The words were printed on individual index cards that were distributed to the players.

#### 2.1.3 Design and procedure

Participants took turns producing non-linguistic vocalizations and oral sounds to express the meanings written on index cards as their partner tried to guess each one. No words or body movements were allowed. Players could see each other and were permitted to interact freely, as long as the vocalizer did not use words or gestures (see demonstration video in the electronic supplementary material).

Each player was given a set of 12 shuffled cards, which always included both items of an antonymic pair. Six meanings held by each player were possessed uniquely by that player, and the remaining six were possessed by both players.

The game was played for 10 rounds. In each round, the first player attempted to communicate the meanings contained on all of their cards, one at a time (i.e. a turn). The players then switched roles. Players shuffled their cards before each round. A turn lasted for up to 10 s, timed by the experimenter. During this time, players were permitted to make as many sounds and guesses as they wished. The experimenter monitored the game, and when necessary, reminded participants not to gesture if they began to move their hands, as well as not to use words if they produced a vocalization that sounded too word-like. The turn ended when the allotted time expired, or when a correct guess was made, which was immediately noted by the vocalizer. If no correct guess was made, then the experimenter called time at 10 s, and the vocalizer shared the correct word with their partner.

Vocalizations were recorded by a lapel microphone attached to the vocalizer and guesses by a flat boundary microphone placed in front of the guesser. Both microphones were plugged into stereo channels of a digital recorder, which recorded the experiment at a sampling rate of 44.1 kHz. The microphones were switched each time the players traded roles.

### 2.2 Analysis

#### 2.2.1 Statistical analyses

Statistical analyses with mixed-effect models were conducted using the lme4 package in R. Significance tests of continuous outcomes were calculated using *χ*^2^-tests that compared the fit of mixed-effect models with and without the factor of interest on improvement in model fit. Significance tests of binomial outcomes used the *z*-values associated with the logistic mixed-effect models. Across all studies, the mixed-effect models included participant and meaning as random effects.

#### 2.2.2 Acoustic measurements

Acoustic measurements were made with Praat phonetic analysis computer software [46]. The onset and offset of each sound was marked in a textgrid. The sound files and textgrids were then fed into a script that automated measurements of the acoustic variables: duration, harmonicity (i.e. harmonics to noise ratio), intensity, pitch and pitch change. For all analyses, the acoustic properties of multiple sounds produced during a single turn were averaged together (i.e. vocalizers contributed one, possibly averaged vocalization per meaning per round), and normalized for each individual speaker.

#### 2.2.3 Measuring stability

We measured the stability of vocalizations across rounds by using the five acoustic measurements to compute the Euclidean distance of each vocalization to the vocalization produced by the same participant for the same meaning in the previous round (only computable for rounds 2–10). The variables were first normalized by participant, and the distance was calculated as the square root of the sum of the squared differences between each variable. To test whether this distance decreased over rounds, we constructed a linear mixed-effect model predicting distance between round *t* and round *t*−1 with round as a fixed effect and speaker and meaning as random factors.

#### 2.2.4 Comparison to target words

To ensure that participants were not simply mimicking the prosodic properties of the corresponding target words with their vocalizations, we compared the vocalizations to the acoustic properties of the actual words. We asked three native English speakers to ‘pronounce the words in a neutral tone of voice’. The speakers—students from the University of Wisconsin-Madison—were naive to the hypotheses of the study. Speakers were recorded pronouncing the words as they were presented in randomized order in a Microsoft PowerPoint presentation. The duration, intensity, harmonicity, mean pitch and pitch change were measured for each pronunciation, normalized by speaker, and averaged for each word. This characteristic set of acoustic properties for each target word was then compared with the charades vocalizations.

### 2.3 Results

**Figure 1.**
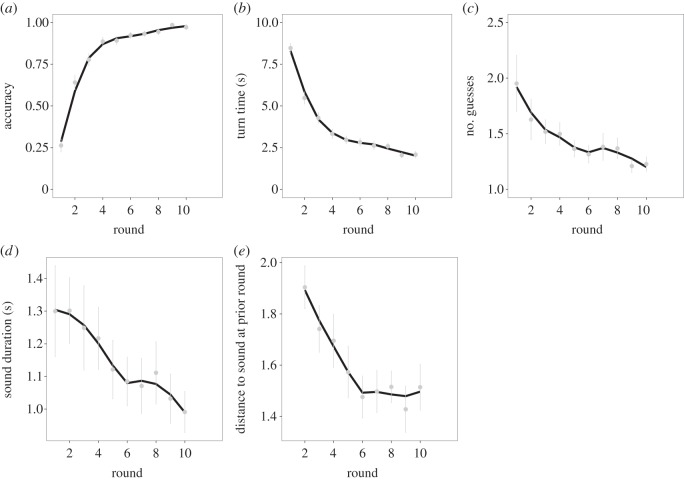
Performance and change in the form of vocalizations over 10 round of vocal charades. The lines in each plot were fit by local regression. Error bars represent standard error between subjects. Round is displayed on the *x*-axis of each plot. (*a*) Mean accuracy increased across rounds. (*b*) The mean number of guesses per turn decreased across rounds. (*c*) Mean turn time (s) decreased across rounds. (*d*) Sound duration (s) decreased across rounds. (*e*) The mean Euclidean distance between the form of sounds produced at round *t* and round *t*−1. Sounds became increasingly similar across the first 5 rounds.

We first examined whether people became more accurate and efficient over the course of the game, i.e. from round 1 to 10. Overall accuracy as proportion of items correct was 82.2%. Figure 1*a* shows that accuracy increased over rounds and eventually exceeded 95% (*z*=17.77, *p*≪0.001, *b*=0.63, 95% CI=[0.57,0.71]). Shown in figure 1*b*,*c*, both turn time (*χ*_1_^2^=702.98, *p*≪0.001, *b*=−0.57, 95% CI=[−0.60,−0.52]) and the number of guesses per turn (*χ*_1_^2^=83.79, *p*≪0.001, *b*=−0.06, 95% CI=[−0.076,−0.050]) decreased over rounds.

We next examined how the vocalizations that participants produced changed over the rounds of the game, specifically whether they became abbreviated and more stable in form. Figure 1*d* shows that the duration of the sounds decreased across rounds (*χ*_1_^2^=24.84, *p*<0.001, *b*=−0.037, 95% CI=[−0.052,0.023]). The vocalizations also became more stable in form across the early rounds of the game, differing less from one round to the next, as shown in figure 1*e* (*χ*_1_^2^=26.86, *p*≪0.001, *b*=−0.050, 95% CI=[−0.069,−0.031]).

**Figure 2.**
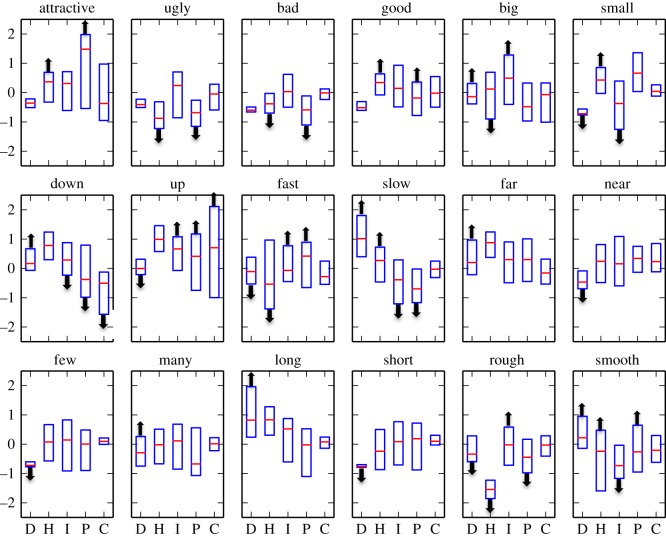
The plots show the acoustic characteristics of each of the 18 meanings. The five variables are represented on the *x*-axis: D, duration; H, harmonics to noise ratio; I, intensity; P, pitch; C, pitch change. All values are normalized (*z*-scored) for each of the five measures. The red line shows the median and the blue box spans the first and third quartiles. The up and down arrows indicate variables that differed reliably between antonymic meanings. For example, vocalizations for *bad* differed from those for *good* by having a lower harmonics to noise ratio and pitch. The variables marked with arrows were the basis for the iconic template of each meaning.

Were the vocalizations that participants produced iconic? To answer this question, we first looked for similarity in the acoustic properties of the vocalizations produced by different participants with respect to each meaning. We reasoned that if participants produced vocalizations according to a sense of iconicity—potentially highly abstract—between their voice and the meanings they expressed, then their vocalizations would take on a similar form for each meaning. Figure 2 shows profiles of the median acoustic characteristics (duration, harmonicity, intensity, pitch and pitch change) of the 18 meanings. The arrows in the figure show the results of logistic-regression models predicting each word versus its opposite with the five acoustic variables as predictors. Notably, each meaning varied reliably from its opposite in at least one acoustic property (with *α*=0.001). We further tested whether the acoustic profiles of each meaning reliably distinguished between all other meanings. We constructed 153 logistic-regression models for all of the meaning pairs (e.g. *up* versus *rough*, *short* versus *few* and so on), of which 132 could be fit with a convergent solution. At *α*=0.05, all but two pairs (98.5%) could be reliably distinguished by at least one significant variable (table 1). In total, we tested five variables for each of the 132 comparisons for a total of 660 tests, and 376 (57.0%) of these differed reliably between meanings (compared to the null prediction of 0.05×660=33). These results indicate that each meaning tended to occupy a unique place in the acoustic space.

**Table 1.** Number of acoustic variables that reliably predicted one meaning versus another. The columns refer to the number of significant variables per comparison from 0 to 5 and the rows to different *α*-values. % significant indicates the percentage of 132 pairs×5 variables=660 tested variables that showed a reliable difference between word pairs. For example, at *α*=0.05, six pairs of meanings differed along all five variables, while only two did not exhibit reliable differences with respect to at least one variable. In total, 57.0% of the 660 tested variables were significant by this criterion.

	no. significant variables per comparison						
*α*	0	1	2	3	4	5	% significant
0.05	2	13	33	45	33	6	57.0
0.01	7	31	43	35	15	1	43.5
0.001	18	46	47	17	4	0	31.4

Finally, to determine that participants were not simply mimicking prosodic properties of the target words, we compared the acoustic profiles of each meaning to the profile of its target word. Across the 18 meanings, we tested the correlation between the average normalized acoustic properties of vocalizations and the properties of the corresponding word. None of the correlations for the five variables showed a reliable relationship between the vocalizations and corresponding words for each meaning (*r*'s<0.40, *p*'s>0.10), confirming our impression that participants were indeed generating non-linguistic vocalizations.

## 3. Charades playback experiments

Iconicity is theorized to ground the creation of a symbol system both by facilitating the generation of novel forms, and also by enabling a perceiver to gain a sense of meaning with limited previous learning. The results of the charades games suggest that people are able to generate iconic vocalizations. We next examined the iconicity of the vocalizations by testing the ability of naive listeners to infer their meaning (cf. [16]). Unlike the charade players whose accuracy was assisted by feedback, repetition and possibly by inadvertent cues like facial expressions and gestures, the only source of information for our naive listeners were the sounds themselves.

### 3.1 Methods

#### 3.1.1 Participants

Four hundred and forty participants were recruited through Amazon Mechanical Turk and they were restricted to be in the USA.

#### 3.1.2 Stimuli

The stimuli were adapted from the recorded vocalizations produced during the vocal charades game. These included vocalizations for each of the 18 meanings from rounds 1, 5 and 10. A random subset of 6 to 9 vocalizations was used for each meaning from each round, for a total of 359 stimuli. Only the first vocalization produced for a trial was used. In some cases, the partner's guessing overlapped with the vocalization. This segment was trimmed off when possible, but if it occurred too early to be trimmed, the vocalization was not used.

#### 3.1.3 Design and procedure

The experiment was conducted in two batches. Batch 1 (*n*=232) tested eight meanings (*big*, *small*, *down*, *up*, *fast*, *slow*, *long*, *short*), and batch 2 (*n*=208) tested the remaining 10 (*attractive*, *ugly*, *bad*, *good*, *far*, *near*, *few*, *many*, *rough*, *smooth*). In both, participants listened to 10 randomly drawn target sounds and selected from 10 options—the eight target meanings plus *attractive* and *ugly*in batch 1 and the 10 possible meanings in batch 2. Across trials, it was possible that participants heard multiple stimuli intended to express the same meaning, but no specific sound was repeated more than once.

**Figure 3.**
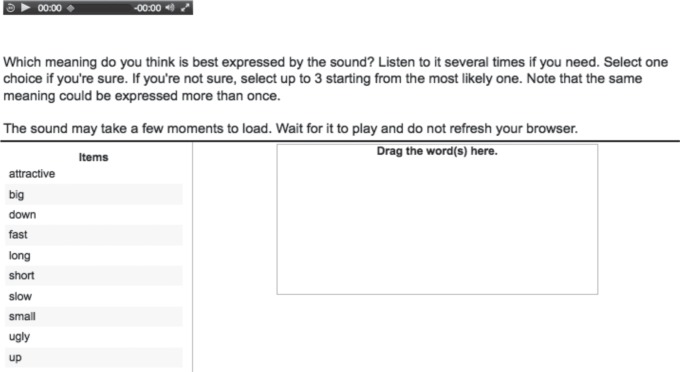
Screenshot of the interface from the playback experiments. Participants listened to a sound as many times as they wanted by clicking on the ‘play’ arrow. They then made their selection by dragging the chosen word into the empty box.

Figure 3 shows the interface of the experiment. Participants could listen to the sound multiple times and made their choice by using the mouse to drag the selected word into the response box. They were forced to make at least one selection, but could make a total of three ranked selections if they were uncertain. We report only their first selection.

### 3.2 Analysis

#### 3.2.1 Word similarity norming

While our main accuracy analyses code accuracy in a discrete way (correct/incorrect), not all errors are the same. For example, confusing ‘attractive’ and ‘good’ may be thought to be a smaller error than confusing ‘attractive’ and ‘far’. To obtain this more continuous error measure, we collected meaning-similarity ratings from a new group of participants. For each of the unique word-pairs, 10 participants indicated the degree to which they were ‘completely different in meaning’ versus ‘almost identical in meaning’ (1–7 Likert scale). Analyses with this continuous error measure mirrored all the results we obtained with the discrete measure. The results depicted in figure 5 rely on the continuous error measure to sort the rows and columns by rated semantic similarity.

#### 3.2.2 Median, iconic and target word distance

To assess the role of iconicity in people's ability to accurately map the vocalization to its intended meaning, we computed two distance measures for each vocalization used as a stimulus (computable for 345 of the 359 stimuli; the remaining 14 lacked a measurable pitch). The first measure was a *median distance* baseline. We created a template for each meaning that consisted of the median values (across participants) for the five acoustic variables. The median distance of a stimulus was its Euclidean distance from these five median values. We measured *iconic distance* by creating a template for each meaning that included only the acoustic variables that differed reliably (*p*<0.01) between antonymic words (figure 2). In this case, instead of the median values, the template consisted of either the maximum or the minimum value (corresponding to the direction of the comparison of the meaning to its antonym). The iconic distance between a stimulus and a given meaning was then computed as the Euclidean distance between the stimulus and the template with respect to only these particular variables.

We also wanted to determine whether the degree to which a vocalization resembled the acoustic properties of its corresponding target word would influence guessing accuracy. Using the template constructed for each target word for the charades game analysis, we calculated the absolute difference between the vocalization and the word for each of the five acoustic variables. We also computed a composite Euclidean distance between the vocalization and corresponding word template.

### 3.3 Results

**Figure 4.**
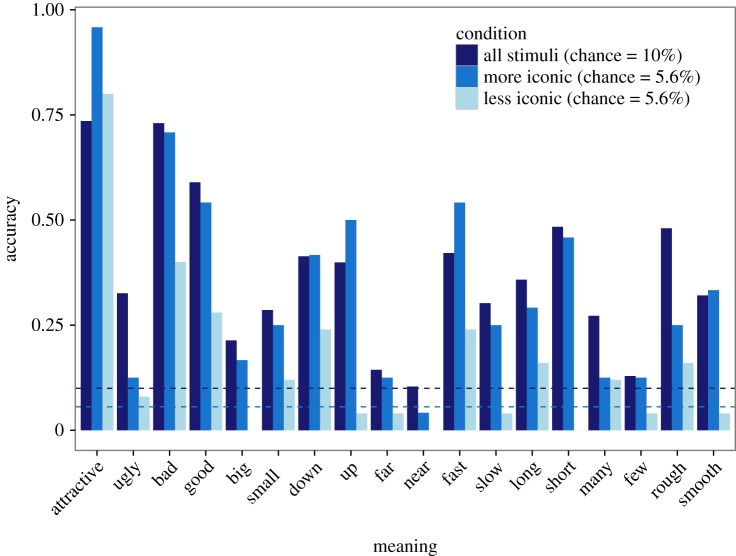
Accuracy of naive listeners at selecting the correct meaning in the playback experiments. The dark blue bars represent playback for stimuli with varied iconicity (10 choices for each meaning; chance=10%, indicated by the dark blue dashed line). The medium and light blue bars show performance from a follow-up experiment with more and less iconic instances of the vocalizations, respectively (18 choices for each meaning: chance=5.6% indicated by the medium blue dashed line). Accuracy was higher for the more iconic versus less iconic vocalizations for all 18 of the tested meanings.

**Figure 5.**
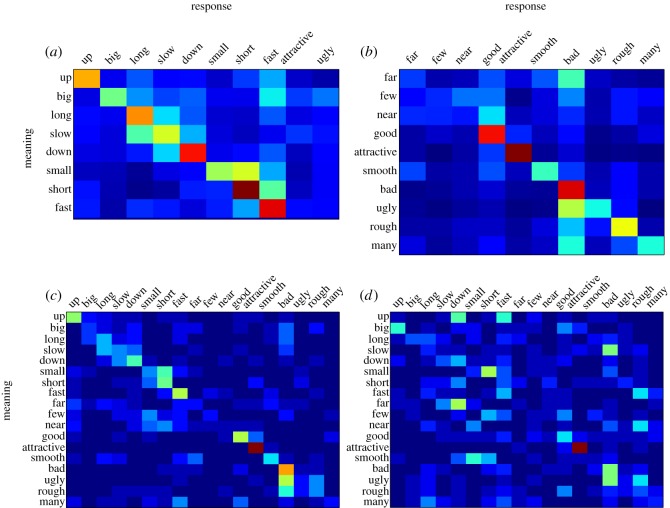
Confusions made for each of the meanings during the playback experiments. The *y*-axis displays the meaning of the sound that was presented, and the *x*-axis displays the meaning that was selected. The ordering of meanings in each matrix was determined by a norming experiment that collected similarity ratings between each possible pair of meanings, with more similar words placed closer together. Warmer colours indicate more frequent choices. (*a*) Results from the first batch and (*b*) from the second batch in the playback experiment. Warmer colours gravitate along the diagonal, showing that listeners tended to select the correct response or a meaning that was similar to it. The results from the follow-up experiment with more and less iconic stimuli are shown in (*c*) and (*d*), respectively. Warmer colours gravitate along the diagonal for more iconic stimuli, but responses for less iconic stimuli appear more random.

**Figure 6.**
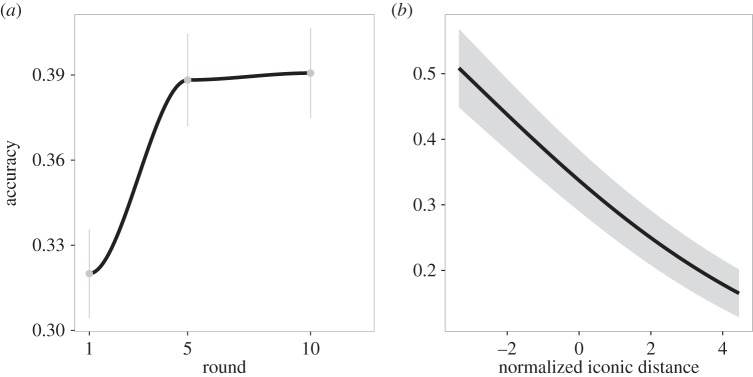
(*a*) Accuracy in the playback experiment as a function of the round in which the stimulus was produced in the vocal charades game. Error bars display standard error between subjects. Accuracy is higher in rounds 5 and 10 compared to round 1. (*b*) Accuracy as a function of the normalized iconic distance of the stimulus. Iconic distance is the Euclidean distance between the vocalization and the iconic template for its intended meaning. Accuracy is higher for vocalizations that are closer to the template.

Compared to a chance rate of 10%, overall accuracy at guessing the intended meaning was 35.6% in the first batch and 37.3% in the second (with no reliable difference between batches, *z*=−0.02, n.s.). Accuracy across meanings ranged from 10.4% (‘near’) to 73.6% (‘attractive’), and 15 of 18 words exceeded 20% (figure 4). These accuracies were strongly correlated with accuracies in the charades game for the same meanings (*r*=0.57, *p*=0.01). Figure 5*a*,*b* shows confusion matrices associated with the 18 different meanings. Accuracy was higher for vocalizations made in rounds 5 (38.8%) and 10 (39.1%) compared to round 1 (32.0%; *z*=3.28, *p*=0.001, *b*=0.03, 95% CI=[0.013,0.052]; figure 6*a*).

The relatively high degree of accuracy by naive listeners indicates that they were able to extract information about meaning from the acoustic signal alone. But is it possible to identify the particular iconic properties that accounted for this ability? To determine this, we compared the *iconic distance* between the vocalization and the template to the non-iconic, *median distance*. Iconic distance was a reliable predictor of guessing accuracy (*z*=−5.71, *p*≪0.001, *b*=−0.20, 95% CI=[−0.27,−0.13]) when controlling for median distance; median distance was not a reliable predictor when controlling for iconic distance (*z*=−1.31, n.s.). As shown in figure 6*b*, listeners were more likely to guess the meaning of vocalizations that had forms closest to the idealized meaning template (i.e. were most iconic by this measure). The addition of *target word distance* as a predictor in the model did not alter these results in any significant way. Target word distance was not a reliable predictor of accuracy (*z*=1.62, n.s.), and iconic distance remained highly reliable (*z*=−3.22, *p*=0.001, *b*=−0.21, 95% CI=[−0.21,−0.05]). With one exception, the same pattern of results was also found for the absolute distance between vocalization and word with respect to each variable separately: iconic distance remained highly reliable (*z*'s<−4.02, *p*'s<0.001), and shorter variable distances did not predict greater accuracy (*z*'s>0.59). The exception was that the distance between the intensity of the vocalization and the target word was a reliable predictor of accuracy, such that smaller distances lead to greater accuracy (*z*=−2.66, *p*<0.01, *b*=−0.15, 95% CI=[−0.26,−0.04]). However, iconic distance remained a highly reliably predictor (*z*=−6.07, *p*<0.001, *b*=−0.22, 95% CI=[−0.29,−0.15]). It is not clear what to make of this intensity result, particularly as words do not typically have a characteristic intensity. Overall, it appears that participants were making use of iconic properties of the vocalizations to infer their meanings, as the vocalizations did not resemble the corresponding English words.

**Figure 7.**
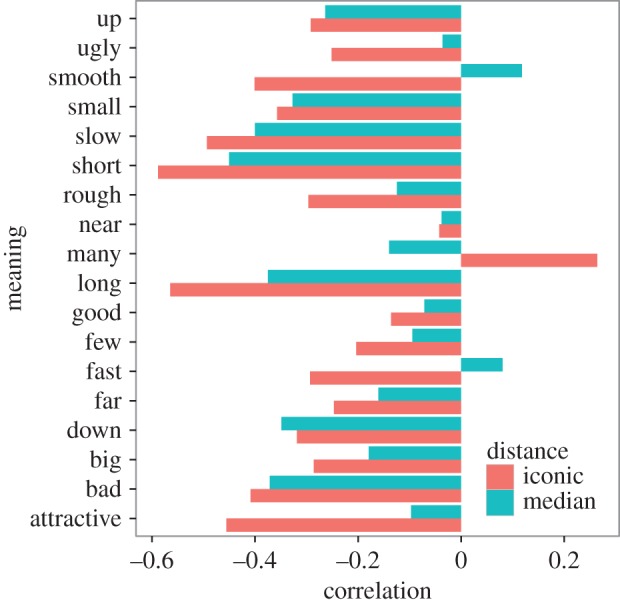
Correlations between the two distance metrics to each particular meaning and probability of selecting that meaning. The correlation coefficient is plotted on the *x*-axis and meaning on the *y*-axis. Red represents iconic distance, and blue median distance. Negative correlations indicate that smaller distances to a particular meaning are associated with a higher likelihood of selecting that meaning. All meanings but *many* are associated with a negative correlation.

Across all meanings, there was a moderate negative correlation between the iconic distance to that meaning and its probability of selection, regardless of its correctness (*r*=−0.30, *p*<0.001), and a weaker negative correlation between selection probability and median distance (*r*=−0.18, *p*<0.001). Stimuli with acoustic properties that were more similar to the iconic template for a particular meaning were more likely to elicit that meaning as a response. Similarity to the non-iconic median template was less predictive of participants' responses. Figure 7 shows the correlations between both iconic and median distance with the selection probability for each meaning. The iconic distance correlation was negative for 17 of 18 meanings and weaker than median distance for only two of the meanings.

## 4. Playback with more versus less iconic vocalizations

We further wondered whether we could identify exemplars of highly iconic vocalizations for each meaning and how accurately guessers would perform with these exemplary stimuli. To test this, we used iconic distance to identify the two most and the two least iconic stimuli for each meaning. Then, in a follow-up experiment, we played these to a new set of listeners in a between subjects design.

### 4.1 Methods

#### 4.1.1 Participants

Participants were recruited through Amazon Mechanical Turk and randomly assigned to a *more iconic* condition (*n*=26) or a *less iconic* condition (*n*=28).

#### 4.1.2 Stimuli

The stimuli were drawn from the set used in the full charades playback experiment. For each meaning, we selected the two stimuli with the smallest iconic distance (i.e. highest levels of iconicity) and the two with the largest iconic distance (i.e. lowest levels of iconicity), resulting in 36 stimuli for each condition.

#### 4.1.3 Design and procedure

The design and procedure were similar to the full charades playback experiment. The critical difference was that participants listened to stimuli expressing all 18 meanings and for each vocalization selected a meaning from the complete set of 18 possible meanings, i.e. an 18-alternative forced choice (chance level=5.6%).

### 4.2 Results

Overall accuracy was higher for more iconic (34.5%) compared with less iconic vocalizations (15.6%; *z*=−3.27, *p*=0.001, *b*=−1.57, 95% CI=[−2.56,−0.64]). Figure 4 shows the accuracy for the more (medium blue bars) and less iconic (light blue bars) vocalizations for each meaning. Figure 5*c*,*d* shows the corresponding confusion matrices.

## 5. Discussion

Our goal was to examine people's ability to generate a simple vocal symbol system through the process of iconic creation, comparable to the process by which many gestural communication systems are known to be generated. We found that participants were successful at communicating a set of 18 different meanings in a vocal charades game. Over 10 rounds, their performance became highly accurate and increasingly efficient. As participants’ vocalizations became more communicatively effective over rounds, they also became shorter and more stable in form. These findings show that people can create novel, non-linguistic vocalizations to express a range of meanings, and that even after relatively few interactions, these vocalizations begin to conventionalize into a system of symbols.

Although some theorists have made strong claims concerning the limited potential of iconicity in the vocal modality (e.g. [18,27,28]), our results demonstrate how people are able to make use of iconicity to ground successful vocal communication—for at least the present set of basic meanings. Tasked with communicating using novel, non-linguistic vocalizations, charades players were inclined to create vocalizations with highly consistent acoustic properties for each particular meaning. This shows that they shared intuitions for how the qualities of their voice mapped to each meaning. In turn, the naive listeners of the playback experiments displayed a considerable degree of accuracy in their ability to interpret the meanings of these vocalizations without any prior learning, showing that they shared intuitions for what vocalizations expressing different meanings *should* sound like. When we quantified the iconic properties of the vocalizations used in playback as a distance metric from iconic templates constructed for each meaning, we found that this measure (iconic distance) predicted accuracy and also the specific meanings that listeners selected in response to the vocalization. In a subsequent experiment, when participants were presented with more iconic stimuli, they were much better at inferring the meanings of sounds than when presented with stimuli having lower iconicity.

Strengthening the case for iconicity, we showed that the vocalizations that participants produced were generally not influenced by the corresponding English words. The forms of the vocalizations were not correlated with the forms of the words for any of the measured variables, and listeners' responses were also not influenced by the degree of similarity between the vocalization and its corresponding word in the playback experiments. It is worth emphasizing that our analysis focused on prosodic properties of the vocalizations, and with the exception of duration, and to some extent pitch, these properties are not characteristically associated with lexical forms in English (e.g. words are not distinguished by the intensity at which they are spoken). Thus, the evidence indicates that participants were able to successfully perform the task of creating non-linguistic vocalizations, which has been a point of concern in other semiotic experiments [45].

Even though participants were able to generate non-linguistic vocalizations, the constraint to communicate vocally without using English may nevertheless have affected the vocalizations they produced. For example, participants may have been pushed to make greater use of gross prosodic features rather than more detailed ‘phonetic’ articulatory gestures that they would associate with speaking English. This reduction of the articulatory space could have driven participants to produce more similar vocalizations than they would have with the full space at their disposal. At the same time, the reduction of articulatory space also limits some potential for iconicity, for instance, reducing the potential for sound symbolism associated with English phonological segments.

According to the process of iconic creation, communicators create iconic signals, which then become conventionalized and moulded into a grammatical system over repeated use and transmission.^3^ Early researchers of signed languages noted the ways in which the iconicity of signs faded over time as the forms became more regularized and systematic, leading to the idea that iconicity appears in the original creation of signs and then decays in a unidirectional process as systematicity sets in [4,21]. Yet, although signed languages exhibit more arbitrariness as they mature, they nevertheless continue to maintain high levels of iconicity [12,15]. We do not know what would happen to the iconicity of signed languages over the much longer time periods over which spoken languages have developed, i.e. tens or hundreds of years compared to tens of thousands of years or more. Additionally, studies of other communication systems suggest that the transition from iconic to more arbitrary signals varies between different kinds of media and interactional contexts [16,30].

Here we found that naive listeners were more accurate with vocalizations generated during later rounds of the charades game, suggesting that the vocalizations became more iconic over rounds, even as they showed evidence of increasing conventionalization. This finding may appear somewhat contrary to the theory of iconic creation and the idea that iconicity facilitates the initial generation of signals. One possible explanation relates to the point that communication involves negotiating an effective signal between interlocutors. The vocalizer's intuition of an iconic vocalization may differ somewhat from that of her partner, and a more widely interpretable iconic vocalization may be established between them over the course of multiple interactions. A second explanation relates to the possibility that the particular iconic properties of vocalizations might have become enhanced over rounds, as less relevant properties were de-emphasized. Such a process of maintaining properties of iconic contrast while relaxing other properties could also facilitate contrasts within the wider system of items—promoting better guessing accuracy between the alternatives by a naive listener.

An additional factor that is likely to affect the balance between iconicity and arbitrariness is the crowding of the semantic space in the communication system. Arbitrariness may increase as larger lexicons put pressure on signal systems to be efficient while maintaining discriminability between meanings [48]. If true, tasking people to differentiate just these 18 meanings may not have posed sufficient crowding of the semantic space to disrupt the iconicity that was originally introduced into the system. It is also possible that if we had run the game for more rounds, the iconicity of the vocalizations and their interpretability to naive listeners may have eventually begun to decline.

One notable qualification of our findings is that, while participants produced *non-linguistic* vocalizations, it is unavoidable that their vocalizations were influenced by other sound–meaning correspondences shared in their culture. In a few cases, they may have been influenced by iconic vocal emblems: for example, a high-pitched whistle for attractive, or a disgusted ‘eww’ for ugly. Another case, recognized as a common conceptual metaphor of American-English culture, is the use of high pitch for up and low pitch for down [49,50]. On the one hand, evidence indicates that verticality–pitch correspondence arises very early in development [35], and scholars have posed plausible accounts of iconic motivation to explain it. For example, small things that make high-pitched sounds tend to be located upward, whereas big things that make low-pitched sounds tend to be down on the ground, and additionally in production, low-pitched vocalizations resonate in the chest, whereas high-pitched vocalizations resonate higher in the head [51]. However, on the other hand, despite these possible explanations, there is considerable variation across languages in how speakers describe and conceptualize ‘high’ and ‘low’ pitches [50]. Farsi speakers use words translating to ‘thin’ and ‘thick’, for instance. These examples illustrate how conventionalized vocal expressions can reflect iconic motivations without being determined by them. While it is not possible to isolate the role of culture here, future work might conduct similar experiments with people across different cultural and linguistic backgrounds to better understand its role in the generation and interpretation of iconic vocalizations.

Another point of qualification concerns the extent to which these findings might generalize to other meanings beyond the present set. Indeed, we found that for a few of the 18 items examined here—particularly *few*, *near* and *far*—the ability of naive listeners to guess their meaning was quite limited and did not exceed chance performance. (For comparison, however, it is worth noting that when non-signing English speakers viewed undisputedly iconic ASL signs and attempted to guess their meanings in a five-alternative multiple choice format, they were no better than chance for the great majority of signs [52].) Although guessing accuracy for playback was low for these meanings, on the production end, charades players were consistent in using the duration of their vocalizations to distinguish *few* from *many*. Specifically, they tended to produce contrasting sequences of repeated sounds—a transparently iconic strategy also used to a lesser extent for *fast* and *slow*. In the case of *near* and *far*, the difficulties may have resulted from the spatial nature of the concepts, which may not easily map to iconic actions of the vocal tract. Yet again, from the perspective of production, charades players' use of longer duration for *far* is intuitive, mapping on to the relative extent of the distance. So it does not appear to be the simple case that these particular meanings are intractable to representation with iconic vocalizations.

Nevertheless, it is important for future studies to consider more broadly the kinds of meanings and conceptual domains that are most readily translated into iconic vocalizations, especially in comparison to gestures. Studies of ideophones across different spoken languages find that they typically express adjectival and adverbial properties [53], and experimental studies have found that people are better at using novel vocalizations to communicate emotion words (e.g. *tired, pain*) compared to actions (*fleeing*, *sleeping*) and objects (*rock, fruit*) [19,30]. Participants' success with the set of meanings tested here—concentrating on various adjectival and adverbial properties—is in line with these previous findings, as is participants' relative success with meanings bearing emotional qualities (e.g. *attractive, bad*). Clearly, some meanings lend themselves more to iconic vocalizations than others, and not just any meaning at all is easily expressed through an iconic vocalization. If participants had not been limited to vocal communication, it is also likely that they would have opted to express some items with gestures or with multimodal combinations of vocalization and gesture [19]. In combination with gestures, iconic vocalizations could function effectively to distinguish different meanings without needing to be as distinctive from each other, since contrasts between the forms of confusable meanings could be distributed across the modalities.

This differential affordance for iconic representation has important implications for understanding the creation and development of unbridled communication systems, like spoken languages. Communication with spoken language involves a coordinated mix of vocalizations with manual and other bodily gestures [54,55]. Given the traditional linguistic principle of the arbitrariness of the sign, many scholars have maintained that, in these systems, the vocal channel primarily functions to carry the arbitrary linguistic components of a message, while the visible gestural channel conveys the iconic, non-linguistic components [55]. Stemming from this idea, some have proposed that spoken languages must have originated as iconically grounded systems of manual and other visible bodily gestures, and at some point in human history, arbitrary vocalizations were bootstrapped on inherently iconic manual gestures [12,19,27,28].

Alternatively, we argue for a more balanced hypothesis of how spoken languages are created from iconic origins, taking into account the combined evidence of sound symbolism, ideophones and other iconic vocal phenomena [14,18,34], as well as this work showing that vocal symbols can be formed through iconic creation. We observe that when humans are free to communicate across modalities, they are inclined to embed iconic form–meaning correspondences in vocalizations as well as gestures. Thus, when facing a naturally occurring challenge to devise a communication system, people are likely to take advantage of the strengths of iconic representation in each modality. As a simple example, they may rely predominantly on gestures for representing actions and spatial relationships, and on vocalizations for representing objects and events that are associated with distinctive sounds. The ability to construct reinforcing representations across modalities—for instance, combining a gesture and vocalization to distinguish a particular predator—could also be advantageous, enabling robust communication over different perceptual conditions (e.g. low lighting, background noise, occluded line of sight). An interesting line of research will be to examine how people develop symbol systems when they are permitted to vocalize and gesture freely, including how their use of each modality changes over repetition and transmission.

## 6. Conclusion

In a variation of a classic thought experiment, Tomasello [28] asked readers to imagine two groups of children, well cared for, but lacking a language model: one group is constrained to communicate with gesture, the other with vocalization. Citing evidence from signed languages and home sign systems, he suggested that the children would naturally use gestures to create a language because of their iconic potential to ground meaning by pointing at things in the world, pantomiming actions, depicting shapes and spatial layouts, and so on. By contrast, he noted that the iconic potential of vocalization is trivial. It is difficult to imagine the children ‘inventing on their own vocalizations to refer the attention or imagination of others to the world in meaningful ways—beyond perhaps a few vocalizations tied to emotional situations and/or a few instances of vocal mimicry…and so the issue of conventionalizing already meaningful communicative acts never arises' [[27], p. 228]. It is unlikely that we will ever have the opportunity to witness the creation of a spoken language, or that we will succeed in the reconstruction of the original spoken languages, which are speculated to have emerged at the start of the Upper Paleolithic (approx. 50 000 years ago), if not long before [10]. But here, by bringing a version of this thought experiment into the laboratory (albeit with UCSC undergraduates in place of language-less infants), we find that vocal symbol systems can, at least in principle, originate from the same process of iconic creation and conventionalization that has been observed in the formation of signed languages and other communication systems.

## Footnotes

1 A compelling case can be made that spoken languages are multimodal systems (e.g. [10,11]), and we consider this important point in Discussion of how vocalizations and gestures might function together in the creation of multimodal systems.

2 We use ‘iconic’ as a modifier of ‘gesture’ independently of whether the gesture co-occurs with speech, or whether the gesture is conventionalized (e.g. an ASL sign). That is, ‘iconic’ refers to the motivated quality of a signal, and ‘gesture’ to its visible modality.

3 Arbitrarily generated signals also become systematic over repeated use and transmission [45,47]; this part of the process is not particular to iconic signals.

## Data accessibility

We cannot share the original vocalizations because they contain potentially identifying information. We have made our analysis and code, including the measurements of the vocalizations, available through the Dryad Digital Repository at the following link: https://datadryad.org/resource/doi:10.5061/dryad.vq33n.
